# The ethical dimension of problems faced in general medicine: relationship with moral sensitivity*

**DOI:** 10.1590/1518-8345.4033.3309

**Published:** 2020-08-31

**Authors:** Janaina Cassana Mello Yasin, Edison Luiz Devos Barlem, Jamila Geri Tomaschewski Barlem, Rosemary Silva da Silveira, Graziele de Lima Dalmolin, Gustavo Baade de Andrade

**Affiliations:** 1 Universidade Federal do Rio Grande, Rio Grande, RS, Brazil.; 2 Universidade Federal de Santa Maria, Santa Maria, RS, Brazil.

**Keywords:** Adult Health, Nursing Ethics, Ethic, Moral, Moral Development, Nursing, Saúde do Adulto, Ética em Enfermagem, Ética, Moral, Desenvolvimento Moral, Enfermagem, Salud del Adulto, Ética en Enfermería, Ética, Moral, Desarrollo Moral, Enfermería

## Abstract

**Objective:**

to identify the main ethical problems and how these relate to the moral sensitivity of nurses working in a general medicine ward.

**Method:**

this qualitative, exploratory, and descriptive study was conducted in a university hospital located in the south of Brazil. A total of 18 nurses working in a general medicine ward were interviewed. A semi-structured interview script was used, and data were analyzed using discursive textual analysis.

**Results:**

nurses considered the main ethical problems to include conflicts at the institutional level, situations involving conflicts with patients and/or family members, and conflicts within the staff. The perception of nurses and how they deal with these problems relate to moral sensitivity. Two categories emerged: experiencing ethical problems, and relationship with moral sensitivity.

**Conclusion:**

because of the multidimensional nature of moral sensitivity, it trains and enables nurses to recognize and deal with ethical problems faced in clinical practice so that nurses become able to make fair and prudent decisions, improving the quality of nursing care.

## Abstract (pt)

**Objetivo:**

conhecer os principais problemas éticos e como estes são relacionados com a sensibilidade moral em enfermeiros atuantes em uma Unidade de Clínica Médica.

**Método:**

pesquisa qualitativa, do tipo exploratório-descritiva, desenvolvida em um hospital universitário do sul do Brasil, com 18 enfermeiros atuantes em uma unidade de clínica médica, por meio de entrevistas semiestruturadas ponderadas via análise textual discursiva.

**Resultados:**

verificou-se que os conflitos institucionais, com o paciente e/ou familiar e com a equipe foram elencados como os principais problemas éticos identificados pelos enfermeiros, sendo a percepção e enfrentamento destes relacionados com a sensibilidade moral, compreendendo duas categorias: vivenciando problemas éticos e relações com a sensibilidade moral.

**Conclusão:**

a sensibilidade moral, pelo seu caráter multidimensional, capacita e habilita os enfermeiros para o reconhecimento e enfrentamento dos problemas éticos na prática clínica e contribui para a tomada de decisão justa e prudente, o que repercutirá na qualificação da assistência de enfermagem.

## Abstract (es)

**Objetivo:**

conocer los principales problemas éticos y cómo están relacionados con la sensibilidad moral en los enfermeros que trabajan en una Unidad de Clínica Médica.

**Método:**

investigación cualitativa, exploratoria-descriptiva, desarrollada en un hospital universitario en el sur de Brasil, con 18 enfermeros que prestan servicios en una unidad de clínica médica, a través de entrevistas semiestructuradas sometidas a análisis discursivo textual.

**Resultados:**

Se detectó que los conflictos institucionales, así como con el paciente y/o la familia y los conflictos con el equipo se enumeraron como los principales problemas éticos identificados por los enfermeros, siendo que, la percepción y el manejo de los mismos se relacionó con la sensibilidad moral, bajo la órbita de dos categorías: “problemas éticos” y “relaciones con sensibilidad moral”.

**Conclusión:**

la sensibilidad moral, debido a su carácter multidimensional, capacita y permite a los enfermeros reconocer y enfrentar problemas éticos en la práctica clínica y, por lo tanto, realizar un proceso de toma de decisiones justo y prudente, que tendrá un impacto en la calificación de la atención de enfermería.

## Introduction

Nursing care, especially clinical practice, is permeated by an interdependent complex work process, which is inherently an ethical practice in which ethical decisions are made when facing tension, problems and conflicts^(^ 1 ^)^.

Nurses working in a hospital context play an essential role when providing care to clinical patients, especially in terms of decision-making. For this reason, nurses need to be morally sensitive, have knowledge, experience, and dynamism so that conflicts faced in daily practice that are related to a divergence of values, uncertainty regarding decision-making, and struggles in the relationship established with others, do not result in ethical problems^(^ 2 ^-^ 3 ^)^.

Moral sensitivity may be considered a moral and intuitive concept that trains and enables workers to identify the moral component of situations of conflict, make decisions, and manage ethical problems, aware of their role and responsibility^(^ 4 ^)^.

Moral sensitivity in the nursing field may be seen as the ability or capacity of nurses to recognize the ethical and moral dimension of their behavior when making decisions on behalf of patients. Nurses, however, are often unable to identify these dimensions because their knowledge and skills are tested daily in their practice, resulting in ethical problems^(^ 5 ^)^.

International studies^(^ 4 ^,^ 6 ^-^ 7 ^)^report that ethical problems may accrue from everyday situations that are experienced in daily practice and involve: questionable care and therapeutic practices, not asking patients an informed consent before performing procedures, insufficient human or material resources, and unequal treatments. Thus, nurses need to be prepared and sensitized morally to recognize the situation causing a given problem and face it with prudence, considering consequences that may affect all those involved.

Perceiving ethical problems was one of the factors that influenced nurses’ moral sensitivity^(^ 8 ^-^ 9 ^)^ the most. For this reason, it is essential to investigate Brazilian general medicine wards, in which studies addressing ethical problems are not related to moral sensitivity.

Thus, this study was intended to fill in this gap, that is, the need to link moral sensitivity to a perception of ethical problems in clinical settings, considering that moral sensitivity enables nurses to identify ethical and moral issues involving nursing care, promoting ethical and fair decision-making to meet the needs of patients as well as favor their rights and interests.

Therefore, this study’s objective was twofold: to identify the main ethical problems and how they are related to moral sensitivity among nurses working in a general medicine ward.

## Method

This qualitative, exploratory, and descriptive study was conducted in the general medicine ward of a university hospital located in the south of Brazil, which exclusively provides care to patients within the Brazilian Unified Health System. It has 203 beds, 38 of which for general medicine, including the following specialties: infectious diseases, pneumology, neurology, hematology, cardiology, and nephro-urology. This unit has 22 nurses, all public employees tendered by *Empresa Brasileira de Serviços Hospitalares (EBSERH)* [Brazilian Hospital Services Company], governed by the Consolidation of Labor Law with a weekly workload of 36 hours.

The participants were selected using a non-probabilistic convenient sample, selected according to their presence at the study setting and availability to participate at the time of data collection. Eighteen individuals who met the inclusion criteria participated in the study; four nurses were on vacation or sick leave and were excluded from the study.

Inclusion criteria were: having a work contract with the institution and not being replacing other employees’ leave. Exclusion criteria were: not being a nurse or been on vacation, leave, or time-off.

One of the researchers individually collected data in June 2018 during the participants’ working hours in a specific room in the hospital’s premises to ensure privacy. Semi-structured interviews were recorded in a portable voice recorder and lasted 25 minutes on average. The interview script contained close-ended questions to characterize the participants and open-ended questions such as: Please tell me what does it mean to be a nurse in a general medicine ward? What are the main ethical issues experienced in your daily practice? Do you believe that the remaining nurses, staff, and institution, in general, recognize ethical situations in the work environment? What do you consider moral sensitivity to be? Do you use moral sensitivity to make decisions when facing ethical problems? How do you think moral sensitivity influence decision-making on behalf of patients? All the interviews were audio-recorded and transcribed verbatim.

Data were analyzed using discursive textual analysis, which is a methodology used with qualitative data and is intended to produce a new understanding of discourse and phenomena based on three stages: unitization of texts, categorization, and communication^(^ 10 ^-^ 11 ^)^.

In the unitization stage, the interviews were analyzed in detail and fragmented until units of meanings emerged from the reports. Categorization was intended to identify relationships between units of meaning, compare them, and gather similar elements of meaning into intermediate categories, from which two final categories emerged: experiencing ethical problems and relationship with moral sensitivity. A new understanding emerged in the last stage of the analysis, which comprises the description and interpretation of meanings that emerged from the reports, enabling the achievement of a new understanding about the ethical dimension of the problems reported by the workers and how they related to moral sensitiveness.

All ethical aspects pertinent to this study have complied with resolution 466/2012 (Institutional Review Board at the Federal University of Rio Grande No. 88/2018). The participants signed free and informed consent forms and were aware of all elements that composed this study and of the possibility to withdraw at any time. The nurses’ reports are identified by letter “N” followed by the number corresponding to the order of interview (N1 to N18).

## Results

Information provided by the 18 participant nurses shows they were aged between 29 and 44 years old; 14 were women; the highest degree obtained by five nurses were a bachelor’s degree, nine had a specialization, three a multi-professional residency, and one nurse had a master’s degree. Professional experience ranged from four to 19 years, while experience in the general medicine ward ranged from three months to two years.

**Figure 1:**
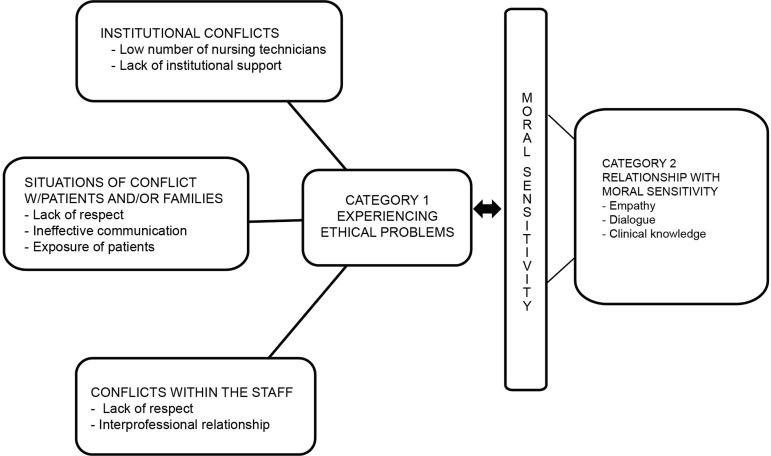
Structural model of how categories emerged. Rio Grande, RS, Brazil, 2018

The categorization process followed the main ethical problems reported by the nurses interviewed in the general medicine ward, which resulted in three elements called “institutional conflicts, situations of conflict with patients and/or family members, and conflicts within the staff.” The three elements defined the intermediate categories so that units of meanings were selected and assigned to each of them. Finally, associating units of meanings resulted in two intermediate categories, namely: experiencing ethical issues and relationship with moral sensitivity. Figure 1 presents the elements that define this study’s categorization process:

Issues concerning conflicts nurses experience in their daily practice compose the category “experiencing ethical issues.” The participants consider that ethical issues may negatively affect the quality of nursing care if left unrecognized or unresolved. Moral sensitivity trains and enables nurses to perceive situations of conflict and make morally appropriate clinical decisions both when dealing with institutional conflicts and conflicts established between staff and patients. Hence, ethical problems recognized by health workers emerged in three dimensions: institutional conflicts; situations of conflicts between workers and family members and/or companions; and conflicts within the team.

Regarding institutional conflicts, the low number of nursing technicians was reported as one of the primary triggers of conflicts related to the organization of work because it leads to work overload, leading to stress and lack of harmony in the workplace: *general medicine wards are large wards and, you know the number of workers (nursing technicians) is undersized. So, there are few workers to meet the demand of patients if you take into account the inpatients’ conditions* (N4); *There are few nursing technicians to assist too many patients (45 patients). Some days there are seven technicians, other days, eight technicians. They get overloaded* (N8)*; The main conflicts refer to the nursing technicians’ heavy workload, they have a very intense workload and get overwhelmed, so the staff ends up quarreling and gets quite stressed out, and even become hostile toward the nurses. It is quite challenging working with the nursing staff we have here* (N11); *It is really tough to do the schedule with a lack of technicians, this is the conflict* (N12).

General medicine wards are facilities that comprise a range of challenges and paradoxes involving care planning based on ethical, rapid, and safe decision-making. Moral sensitivity enables nurses to feel more self-assured when making decisions in the face of ethical conflicts, with a sense of improved leadership and problem-solving capacity.

Lack of institutional support leads nurses to feel helpless; the participants report that even though the institution acknowledges the system’s weaknesses, it does not support workers. Moral sensitivity is needed for nurses to recognize the ethical dimension of their attitudes to prevent their decisions negatively affect nursing care. *The institution sometimes does not heed the problems affecting the nursing care or the workers; it sort of listens to what people say but doesn’t really try to know what is going on. It* [institution] *acknowledges the situation but does not address some issues, for instance, they know there are conflicts, but often overlook the problems, not facing or dealing with them (N6); The institution is not very concerned in emotionally supporting the workers facing conflicts* (N7).

Regarding situations of conflict experienced with patients and/or family members, the participants mentioned a lack of respect on the part of patients and/or their companions and also on the part of workers toward patients. Relationships established in a hospital environment, especially in a clinical setting, are often intense, considering this unit assists patients with varying degrees of complexity both in terms of minimal care and intensive care. Nurses are supposed to be morally sensitive to make decisions and be aware of their roles and responsibilities. *Conflicts with patients and companions (…), sometimes a companion/patient complains of a technician, so I try to understand both the technician and patient/companion* (N6); *Regarding ethical conflicts, we don’t have many of these conflicts here, especially between workers and patients. I don’t know whether it is a cultural thing, but it is very different from the state where I come from* (N11).

Ineffective communication and patient exposure were also listed as situations that lead to conflicts in the general medicine ward with patients and/or family members. Nurses report that insufficient or inappropriate information is provided to patients while patients are sometimes physically exposed. Given a lack of essential equipment, such as screens, the privacy of patients is not always ensured. *I try to be very communicative, I talk (…) because I sometimes witness a lack of communication on the part of my colleagues, but I always talk and clarify things* (N5); *So that the problem is that sometimes there is a lack of screens and it prevents me from respecting the privacy of patients* (N7).

Therefore, clinical nurses experience complex situations, demanding them to be morally sensitive to deal with the fragility of people, whether that of patients or family members. Respecting the autonomy of patients is a fundamental premise for clinical decision-making; thus, these workers should establish effective communication mediated by clear and concise information provided to patients.

Thus, moral sensitivity is developed as nurses provide accurate information and attentively listen to complaints, which strengthens and qualifies workers to recognize the real needs of patients and make ethical and prudent decisions.

Regarding the subcategory conflicts within the staff, the participants report that inter-professional relationships also configure as an ethical problem. The participants report that because a general medicine ward is an environment where individuals from different professions work in the multidisciplinary team, it gives way to the emergence of ethical issues that affect good coexistence within the inter-professional team; competition within the nursing staff; and higher demand on the part of physicians toward nurses, without considering the nurses’ and nursing technicians’ actual responsibilities: *There is much competition, and I think the problem within the general medicine ward is competition among nurses* (N3)*; The greatest problem in my opinion is among the workers themselves, within the staff* (N5)*; Conflicts between teams, because it is a very large staff, so sometimes there are conflicts* (N6)*; The physicians, you know, prescriptions for everything, care provided to patients, sometimes they prescribe so many things and don’t even know what we actually do. It doesn’t matter whether it is our responsibility or not, they put it there and want it to be done, you know. Sometimes we don’t even know how to do something because it is not our job, but it is there and they will demand it* (N16).

According to the participants, conflicts within the staff also emerge because of a lack of respect within the nursing staff itself, both on the part of nursing technicians not acknowledging a nurse as being the head of the unit, and on the part of nurses not mediating and facilitating the relationships within the nursing staff, which may lead to an exhausting, tense, and disharmonious work environment: *There is much conflict within the team (…) there is a lack of respect. If you are a technician, you have to respect nurses. Still nurses, even been hierarchically superior, ought to respect technicians. I think there is a lack of respect within the staff overall* (N4)*; Conflicts within the team, issues we have to deal with when we arrive here and some technicians impose themselves against us, complying with having very few technicians, even though it is not our fault. We try to talk, but some of them get irritated. Sometimes there are even quarrels, but on our part, nurses, we try to keep quiet whenever we have a meeting and try to reach a consensus; otherwise, it becomes a very stressful place to work* (N8); *Conflicts within the staff itself (...) There are many patients to take care* (N10).

Moral sensitivity provides nurses improved ability to face and recognize situations of conflict and implement strategies that improve interactions within the staff, promoting a conducive work environment and ensuring respect and professional autonomy so that stressors taking place in everyday life do not affect the essence of nursing, which is to deliver care. This is especially important in clinical units in which the high demand for service due to the number and profile of inpatients demand workers to make the environment the most harmonious and light as possible.

Hospital facilities, especially clinical settings, are marked by a divergence of values and uncertainty regarding decision-making that concerns institutional conflicts, situations of conflict between the staff and patients and family members, which often result in ethical problems not fully acknowledge in their moral dimension. Thus, nurses need to be morally sensitive to identify the moral component of each situation of conflict and make clinical decisions based on professional ethics and work organization, taking into account the real needs of patients. More sensitive workers are more apt to ensure personal and patient satisfaction and also promote a more harmonious work environment.

The category “relationship with moral sensitivity” reveals that when nurses recognize ethical problems in a general medicine ward, they show they are morally sensitive to make appropriate clinical decisions. Thus, nurses report that empathy, dialogue, and clinical knowledge are related to moral sensitivity.

Regarding essential components that support the resolution of ethical problems concerning institutional conflicts or conflicts between patients and/or companions and the staff, the participants were aware that such ethical problems and the feelings they elicit might significantly impact the care provided to patients. According to the study’s participants, nurses should develop the ability to put oneself on someone’s else place, in order to made clinical decisions when facing ethical problems permeating the care delivered to the inpatients: *We have to see one another, mirror ourselves. Because, sometimes we don’t put ourselves in somebody else’s place, to understand somebody else’s problem, to know why you have a make a given decision, you know?* (N2); *If we put ourselves in the place of a patient, who complaints a lot, but has been hospitalized for three months, you’ll have the ability to sensitize yourself and manage conflicts* (N4); *It is the ability to put yourself in somebody else’s shoes, identify the conflict and try to solve it the best you can* (N10); *I think that whenever you have empathy, you put yourself on somebody else’s place, you manage to guide both your practice and that of your team, you know, guide care delivery* (N13).

Because moral sensitivity sharpens one’s benevolent motivation, morally sensitive nurses act with empathy and integrity, ensuring the rights, privacy, and autonomy of both patients and workers.

Another aspect reported by the participants was the importance of establishing a candid, objective, and sincere dialogue to identify ethical problems concerning conflicts between the staff and patients and family members. Clinical settings are spaces where relationships are intense; hence, nurses recognize that a candid dialogue is an element of moral sensitivity that favors a relationship of trust to be established among teams and between workers and patients and/or families, enabling them to provide clinical care, resolve and minimize situations of conflicts and ethical issues: *Usually, we call the team, we talk and dialogue* (N2); *I invite my colleagues to talk, and may even invite all those involved to speak* (N5); *We always have to remain calm when dealing with conflicts and have a conversation, there is no point in losing your temper. Both parts have to stay calm and talk because it is only through reasoning that things are resolved. Dialogue is the basis of everything, if you have a conversation; you resolve conflicts* (N12).

Finally, the nurses taking part in this study acknowledge that clinical knowledge is essential for professionals to recognize inappropriate behaviors and deal with them without harming patients. Hence, clinical knowledge enhances the clinical and critical view of nurses, making them self-assured to question actions and meet the patients’ real needs. *Moral sensitivity makes me more confident, more self-assured to make the right decisions, and how to discuss with patients* (N3); *Having knowledge regarding recurrent situations in general medicine because then you have a north guiding your decisions, you are aware (...). If a person is morally sensitive and has a more holistic perspective, she or he can see the whole rather than a specific conflict* (N6); *Being focused on the patient to provide dignified care and mainly, having self-knowledge in every situation* (N11); I guess you acquire more experience with practice. So you have a background that helps you to have the courage to take a stand (N13).

## Discussion

Content collected during the interviews shows that nurses consider situations that somehow impede nursing care to be efficiently and efficaciously provided to be ethical issues. A Brazilian study addressing how nursing workers identify ethical problems in clinical and surgical clinical hospitalization wards reports similar findings^(^ 2 ^)^. Moral sensitivity enables nurses to identify ethical problems and minimize moral conflicts, improving the quality of care delivery^(^ 12 ^)^.

The first category “experiencing ethical problems” revealed that nurses identify institutional conflicts, situations of conflict with patients and/or family members, and conflicts within the staff. These findings corroborate those of a study addressing primary health care nurses and the identification of ethical issues, reporting problems concerning management, patients, and within the staff^(^ 13 ^)^. The identification of ethical issues in clinical settings contributes to improving nursing care. Thus, nurses need to be morally sensitive to recognize the ethical dimension of their actions when making clinical decisions^(^ 14 ^)^.

Regarding institutional conflicts, the participants report ethical problems to include: lack of institutional support and an insufficient number of workers. A literature review^(^ 6 ^)^ corroborates these findings concerning ethical dilemmas experienced by nurses during clinical practice. Problems such as lack of organizational support and lack of human and material resources lead nurses to experience ethical issues that require competence in terms of ethics. Hence, when nurses realize they need improved working conditions and do demand such conditions, they are using their moral sensitivity^(^ 12 ^)^.

In terms of situations of conflict established with patients and/or family members, disrespect toward patients, ineffective communication, and exposure of patients configure ethical issues. These findings corroborate with a literature review^(^ 15 ^)^ addressing ethical dilemmas in the nursing field, which highlighted that the main problems include disrespect, ineffective communication, and difficulty ensuring the privacy of patients. Therefore, nurses’ moral sensitivity is a resource that enables them to recognize ethical issues in clinical settings and promote actions based on the clarification of doubts, reassuring patients and minimizing their anguish, ensuring their rights and privacy, and minimizing conflicts arising from such conflicts^(^ 3 ^,^ 16 ^)^.

Another ethical problem this study’s participants listed included conflicts within the staff, mainly characterized by a lack of respect within the nursing team and interpersonal conflicts. One study^(^ 12 ^)^addressing Iranian nurses reports that ethical problems related to interpersonal relationships both within the staff and within the inter-professional team, hinder the development of moral sensitivity that would enable them to cope with dilemmas. Therefore, workers experience discomfort, distress, difficulty in adapting to clinical settings, and disqualification of nursing care delivery^(^ 17 ^-^ 18 ^)^.

The second category “relationship with moral sensitivity” shows that nurses use elements such as empathy, dialogue, and clinical knowledge to deal with and resolve the ethical issues shown by the first category. These findings are in agreement with the results reported by one study^(^ 19 ^)^conducted with Dutch nurses, which reports that nurses’ knowledge, communication, and ability to put themselves in someone’s place when making decisions is an ethical competence used to solve ethical dilemmas in the nursing field. Therefore, moral sensitiveness is an ethical competence required by nurses to plan actions that ensure technical excellence and efficient decision-making to result in personal satisfaction as well as that of patients and staff^(^ 20 ^)^.

Empathy was reported as one of the elements of moral sensitivity that contributes to recognizing and dealing with ethical issues taking place in a general medicine ward. According to a study^(^ 21 ^)^ conducted with nurses and addressing their perceptions of humanized care in a clinical setting, nurses providing hospital care to adult patients should establish empathy and mutual respect within the staff and also toward patients to identify ethical dilemmas. Moral sensitivity is a resource that awakens in nurses a benevolent motivation to do what is good for patients, promoting the identification of ethical problems and making decisions with a greater sense of responsibility, aware of how their actions may affect the staff as well as the lives of patients and their families^(^ 22 ^)^.

Dialoguing was another element of moral sensitivity identified in this study to facilitate the perception and deliberation of moral problems faced in general medicine wards. Similar to this finding, one study^(^ 23 ^)^ addressing nurses to identify their beliefs and actions when advocating for patients, reports that nurses should use effective communication by establishing candid and sincere dialogues to ensure the rights of patients as well as their autonomy. Morally sensitive nurses are more capable of perceiving the real needs of patients and are confident to inform them of their rights in the face of ethical implications, thus advocating on behalf of patients.

Study^(^ 24 ^)^ conducted in Tehran about moral sensitivity reports that nurses need to be morally sensitized to implement clinical knowledge that enables them a critical view to assess and establish care strategies, making decisions that meet the individual needs of patients. These findings are similar to those found in this study. The participants identified that clinical knowledge is related to moral sensitivity when identifying and intervening in ethical problems. The development of moral sensitivity improves nurses’ ability to diagnose and intervene in dilemmas to ensure integral care and patients’ autonomy^(^ 25 ^)^.

Finally, this study’s results show that nurses consider that ethical problems accrue from everyday concerns that take place within nursing care, which may be explained by the fact that workers may not be apt to differentiate routine from ethical conflicts in their practice due to an imbalance of power that permeates nursing practice in which the nurses’ skills and ability to make clinical decision-making are tested every day^(^ 26 ^)^, resulting in underestimating the ethical dimension of care.

Therefore, addressing the ethical challenges that are commonly experienced in professional practice in the curricula of training programs and also in continuing education provided in hospital settings, will train workers to recognize ethical and moral problems and awaken the potential of nurses for moral sensitivity in order to solve problems and meet the real needs of patients^(^ 16 ^)^.

These results are expected to contribute to nursing workers recognizing moral sensitivity as an aspect that enhances their perception and ability to deal with ethical problems, improving their autonomy and self-assurance, supporting reflection and decisions whenever they face ethical issues, and consequently make morally appropriate clinical decision-making.

This study’s limitations include the fact it has a qualitative approach and addressed a specific sample of nurses working in the general medicine ward of a hospital located in the south of Brazil, which prevents generalization of results. Another limitation refers to a lack of Brazilian studies addressing moral sensitivity so that the results reported here cannot be compared to situations experienced by nurses in other Brazilian contexts.

## Conclusion

This study reveals that the participant workers consider ethical problems faced in a general medicine ward to include institutional conflicts, situations of conflict established with patients and/or family members, and conflicts within the staff. Relating these with moral sensitivity showed that the participants employed elements of moral sensitivity such as empathy, dialogue, and clinical knowledge to recognize and deal with problems in their daily practice in order to make clinical decisions based on professional ethics and personal values in accordance with the patients’ real needs.

Thus, the importance of the process of recognizing and dealing with ethical problems is highlighted in nursing practice as well as its relationship with moral sensitivity as it can contribute to strengthening nurses as professionals, developing ethical working environments, and establishing ethical decision-making that benefits patients.

Finally, it is essential to ask whether this study’s results would be similar in different health services and settings. How do nurses in other contexts identify ethical problems and relate them with moral sensitivity? These are helpful questions to support studies addressing moral sensitivity in other Brazilian contexts addressing the managerial, care, and teaching dimensions of nursing.

## References

[1] Mallari MG, Tariman JD (2017). Ethical frameworks for decision-making in nursing practice and research: An integrative review. Via Sapientiae.

[2] Montenegro LC, Rénno HMS, Caram CS, Brito MJM (2016). Problemas éticos na prática de profissionais de saúde em um hospital escola. Av Enferm..

[3] Moreira DA, Ferraz CMLC, Costa IP, Amaral JM, Lima TT, Brito MJM (2019). Professional practice of nurses and influences on moral sensitivity. Rev Gaúcha Enferm..

[4] Lützén K, Dahlqvist V, Eriksson S, Norberg A (2006). Developing the concept of moral sensitivity in health care practice. Nurs Ethics.

[5] Barlem ELD (2018). Sensibilidade moral e formação profissional de enfermagem. Rev Enferm UFSM.

[6] Haahr A, Norlyk A, Martinsen B, Dreyer P (2019). Nurses experiences of ethical dilemmas: a review. Nurs Ethics..

[7] Esmaelzadeh F, Abbaszadeh A, Borhani F, Peyrovi H (2017). Ethical sensitivity in nursing ethical leadership: a content analysis of Iranian nurses experiences. Open Nurs J..

[8] Dalla Nora CR, Zoboli E, Vieira MM (2016). Moral sensitivity and related factors: the perception of nurses. Cogitare Enferm..

[9] Schallenberger CD, Tomaschewski-Barlem JG, Barlem ELD, Rocha LP, Dalmolin GL, Pereira LA (2019). Moral sensitivity components identified among nurses from Intensive Care Units. Rev Bras Enferm..

[10] Moraes R, Galiazzi MC (2011). Análise textual discursiva.

[11] Moraes R, Galliazzi MC (2006). Discursive textual analysis: a multiple face reconstructive process. Ciênc & Educ..

[12] Amiri E, Hossein E, Maryam V, Jafarabadi AM, Hossein AA (2018). Relationship between nurses' moral sensitivity and the quality of care. Nurs Ethics..

[13] Dalla Nora CR, Zoboli ELCP, Vieira M (2015). Ethical problems experienced by nurses in primary health care: integrative literature review. Rev Gaúcha Enferm..

[14] Jamshidian F, Shahriari M, Aderyani MR (2018). Effects of an ethical empowerment program on critical care nurses' ethical decision-making. Nurs Ethics..

[15] Rainer J, Schneider JK, Lorenz RA (2018). Ethical dilemmas in nursing: an integrative review. J Clin Nurs..

[16] Escolar-Chua RL (2018). Moral sensitivity, moral distress, and moral courage among baccalaureate Filipino nursing students. Nurs Ethics..

[17] Herrera MFJ, Axelsson C (2015). Some ethical conflicts in emergency care. Nurs Ethics..

[18] Dalla Nora CR, Zoboli ELCP, Vieira MM (2017). Moral sensitivity in Primary Health Care nurses. Rev Bras Enferm..

[19] Cusveller B, Schep-Akkerman A (2016). Towards a competency assessment tool for nurses in ethics meetings. Nurs Ethics..

[20] Mendonça FAC, Menezes MVM, Amorim SC, Morais FDM, Feitosa EMN, Lacerda CMM (2017). Ethical nursing processes in state of Ceará: reflection for professional practice. Enferm Foco..

[21] Carvalho DO, Santos NNRC, Silva ARV, Carvalho GCN (2015). Percepção do profissional de enfermagem acerca do cuidado humanizado no ambiente hospitalar. R Interd..

[22] Borhani F, Abbaszadeh A, Mohamadi E, Ghasemi E, Hoseinabad-Farahani MJ (2017). Moral sensitivity and moral distress in Iranian critical care nurses. Nurs Ethics..

[23] Tomaschewski-Barlem JG, Lunardi VL, Barlem ELD, Ramos AM, Figueira AB, Fornari NC (2015). Nursing beliefs and actions in exercising patient advocacy in a hospital context. Rev Esc Enferm USP.

[24] Mahdiyoun SA, Pooshgan Z, Imanipour M, Razaghi Z (2017). Correlation between the nurses, moral sensitivity and the observance of patients' rights in ICUs. Med Ethics J..

[25] Tuvesson H, Lützén K (2017). Demographic factors associated with moral sensitivity among nursing students. Nurs Ethics..

[26] Lunardi VL, Lunardi-Filho WD, Silveira RS, Silva PA, Mancia JR (2016). Nursing management and construction of ethical environments. Enferm Foco..

